# Histopathological evaluation of *Senecio rhizomatus* Rusby in 7,12-dimethylbenz(α) anthracene-induced breast cancer in female rats

**DOI:** 10.14202/vetworld.2021.569-577

**Published:** 2021-03-06

**Authors:** Jorge Luis Arroyo-Acevedo, Oscar Herrera-Calderon, Juan Pedro Rojas-Armas, Roberto Chávez-Asmat, James Calva, Tapan Behl

**Affiliations:** 1 Laboratory of Experimental Pharmacology, Faculty of Medicine, Universidad Nacional Mayor de San Marcos, Av. Miguel Grau 755, Cercado de Lima 15001, Peru; 2 Department of Pharmacology, Bromatology and Toxicology, Faculty of Pharmacy and Biochemistry, Universidad Nacional Mayor de San Marcos, Jr Puno 1002, Lima 15001, Peru; 3 Association for the Development of Student Research in Health Sciences, Faculty of Medicine. Universidad Nacional Mayor de San Marcos, Av. Miguel Grau 755, Cercado de Lima 15001, Peru; 4 Departamento de Química y Ciencias Exactas, Universidad Técnica Particular de Loja, San Cayetano s/n, 1101608 Loja, Ecuador; 5 Department of Pharmacology, Chitkara College of Pharmacy, Chitkara University, Punjab 140401, India

**Keywords:** breast cancer, carcinogenic, experimental pharmacology, phytotherapy, *Senecio rhizomatus*

## Abstract

**Background and Aim:**

*Senecio rhizomatus* Rusby (SrR) is a medicinal plant of the Asteraceae family and traditionally consumed as infusion in the Andean region from Peru for inflammatory disorders. This study aimed to determine the histopathological changes afforded by SrR in 7, 12-dimethylbenz[a]anthracene (DMBA)-induced breast cancer (BC) in rats.

**Materials and Methods:**

An ethanolic extract of SrR aerial parts was prepared by maceration with 96% ethanol, and the chemical components were identified by gas chromatography coupled to mass spectrometry; the antioxidant activity was determined by 1,1-diphenyl-2-picril-hidrazil (DPPH) assay; and the acute toxicity was assessed according to the OCED 423 guidelines. In a pharmacological study, 30 female Holztman rats were distributed randomly into five groups, as follows. Group I: Negative control (physiological serum, 2 mL/kg); Group II. DMBA (80 mg/Kg body weight); and Groups III, IV, and V: DMBA + ethanol extract of SrR at doses of 10, 100, and 200 mg/kg, respectively.

**Results:**

The antioxidant activity of the SrR extract against DPPH was 92.50% at 200 mg/mL. The oral administration of SrR at doses of 50, 300, 2000, and 5000 mg/kg did not show any clinical evidence of toxicity or occurrence of death. The groups that received SrR presented a lower frequency of tumors and acumulative tumor volume compared with the DMBA group (p*<*0.05); the DMBA group exhibited a higher incidence of necrosis and moderate mitosis, up to 66.67% and 100.00%, respectively. Finally, infiltrating carcinoma with extensive tumor necrosis was evidenced.

**Conclusion:**

In experimental conditions, the ethanolic extract of SrR had a protective effect in DMBA-induced BC in female rats. Furthermore, the antioxidant activity of its main phytochemicals could be responsible for the effect observed, and SrR seems to be a safe extract in the preclinical phase.

## Introduction

Epidemiological studies of cancer revealed that breast cancer (BC) has a high prevalence in women and, together with lung and colorectal cancer, contributes to more than 50% of all cases of cancer. BC represents 30% of the rate of all new cancer diagnoses, thus being the type of cancer in women and being the second most common main cause of death [1]. In Peru, BC is the second most frequent neoplasm, which has a great economic impact, and has a poor survival rate because of its late diagnosis; thus, the ­establishment of a comprehensive plan that implements strategies for early diagnosis and timely medical treatment of positive cases is necessary to reduce mortality [2].

Alternative and Complementary Medicine (ACM) is part of the folk tradition in several countries and its effectiveness in medical practice is scientifically proven; thus, ACM is rising worldwide. The World Health Organization recommends the incorporation of ACM into national health systems [3,4]. In Peru, a complementary medicine service was implemented in EsSalud (a social health insurance) in 1998 and offered a wide variety of alternative complementary therapies, such as herbal therapy [5].

In Northern Peru, 47 species of plants, mostly *Asteraceae*, followed by *Gentianaceae* [6,7], are traditionally used to manage cancerous conditions. In the American continent, *Asteraceae* is the richest family in species in the Southern region (15%), North America (14%), and Mexico (13%). Moreover, it is the second most diverse plant family in most of the Andean and Central American tropical countries [8]. After the publication of the catalog of flowering plants and gymnosperms, 222 genera and 1432 *Asteraceae* species were registered in Peru; subsequently, the registry was updated to 245 genera and 1530 species, with 14 genera being endemic, that is, *Ascidiogyne*, *Caxamarca*, *Ellenbergia*, *Holoschkuhria*, *Hughesia*, *Nothobaccharis*, *Uleophytum*, *Syncretocarpus*, *Bishopanthus*, *Chionopappus*, *Pseudonoseris*, *Chucoa*, *Schizotrichia*, and *Aynia* [9]. In Lima, the capital of Peru, about 385 species are known, 343 of which correspond to native and/or naturalized species and 42 are exclusively from crops, with the predominant families being *Senecioneae*, *Heliantheae*, *Asteraceae*, and *Inuleae* [10,11].

The *Asteraceae* family comprises species such as *Senecio rhizomatus* Rusby (SrR; Known as Llankahuasi), *Bidens pilosa* L., and *Chuquiraga spinosa* Lessing. The previous studies have indicated that the methanolic fraction of *B. pilosa* stopped the progression of 7, 12-dimethylbenz[a]anthracene (DMBA)-induced BC in rats [12]; moreover, *C. spinosa* also exerted a protective effect against DMBA-induced BC in rats because of its anti-tumorigenic, hypolipidemic, hypoglycemic, antioxidant, and antigenotoxic properties [13].

BC is pathology with a high frequency in women; thus, it is necessary to continue seeking therapeutic alternatives, such as natural products. Thus, the availability of previous studies of plants in the same family may lead to the exploration of their probable effects as antitumor agents. Therefore, the aims of this study were to (1) determine the protective effect of the ethanolic extract of the aerial parts of SrR in DMBA-induced BC in rats; (2) identify its main chemical components using gas chromatography coupled to mass spectrometry GC-MS; (3) determine its *in vitro* antioxidant activity against the 1,1-diphenyl-2-picril-hidrazil (DPPH) radical; (4) to assess its acute toxicity at a single dose according to the Organization for Economic Cooperation and Development (OECD) 420 guidelines; and (5) perform a histopathological study after the administration of SrR to female Holtzman rats.

## Materials and Methods

### Ethical approval

This research was approved by the Ethics Committee at the Faculty of Medicine of the Universidad Nacional Mayor de San Marcos, ID N° 0310 (November 4, 2018). During the study, the specifications proposed by guidelines for the welfare and use of animals in cancer research were followed [14].

### Study period and location

The study was conducted from December 2018 to January 2020 at the Faculty of Medicine, Universidad Nacional Mayor de San Marcos (UNMSM), Lima, Peru.

### Plant material

The aerial parts of SrR were collected from Tambo district, Huancayo (Junín- Peru) at the coordinates 12°03′01″S - 75°13′17″W at 3260 m.a.s.l. in April 2018. A voucher specimen is kept in the Herbarium of the Universidad Nacional Mayor de San Marcos, Peru (Id. 059-USM-2018). The plant was recognized by Hamilton Beltran-Santiago, Biology professor and expert in Botany.

### Extraction procedure

The aerial parts of SrR were dried at room temperature (20°C) and then subjected to successive extractions using 96% ethanol, similar to a procedure previously [15]. The extraction of SrR was completed within 72 h and replicated three times. The extract obtained was concentrated using a rotary evaporator at 40°C and 80 rpm, and finally stored at 4°C in an amber flask until further use.

### GC-MS analysis of the volatile components of S. rhizomatus

The analysis of the extract was performed in an Agilent Technologies Chromatograph 6890 N series coupled to a mass spectrometer-detector (Agilent series 5973) operated in the electronionization mode at 70 eV and fitted with a 5% diphenyl and 95% dimethylpolysiloxane capillary column (DB-5 MS, 30 m × 0.25 mm × 0.25 mm). Helium was used as the carrier gas (1.00 mL/min in the constant flow mode). The injection system was operated in split mode (40:1) at220°C. The GC oven temperature was 40°C, which was kept for 3 min, then increased to 270°C with a gradient rate of 8°C/min. The ion source temperature was 250°C. One microliter of a solution of the extract diluted in CH_2_Cl_2_ (1:100, v/v) was injected into the apparatus. The extract components were identified through a computer search using the Wiley Registry of Mass Spectral Data (6^th^ edition), and through comparison of the calculated linear retention indices with data from the literature NIST.

Quantitative data were obtained from peak areas using a flame ionization detector [16]. The percentage composition of the extract was determined based on the correlation of GC peak areas to the total chromatogram without applying any correction factor [17].

### *In vitro* antioxidant activity against the DPPH radical

The *in vitro* antioxidant activity of the ethanol extract was evaluated using the method of Herrera *et al*. [18]. A methanol solution containing DPPH was used at a concentration of 20 mg/mL, and the extract was used at concentrations of 200, 100, 50, and 10 mg/mL in methanol [18].

The initial absorbance was established at 0.6±0.05 (100% of free radicals; 517 nm).

Scavenging activity (%) = [(A0-A1)/A0] × 100

Where A0 is the absorbance of the control ­reaction and A1 is the absorbance in the presence of the sample, corrected by the absorbance of the sample itself (blank).

### Experimental animals

Female albino Holtzman rats with an average weight of 160±20 g and BALB/c female mice weighing 25-30 g were purchased from the National Institute of Health (INS) of Peru and conditioned for 7 days in large, ventilated cages with standard pelletized food and purified water *ad libitum*. The study was carried out in the facilities of the Faculty of Human Medicine of the UNMSM. The rats were kept in a 12 h:12 h light:dark cycle at a temperature of 21°C±2°C.

### Evaluation of the antitumor effect of the extract on DMBA-induced BC in female rats

#### Experimental cancer induction in female rats

The evaluation of BC induced in female’s rats was performed according to the method of Wang and Shang [19]. A carcinogenic chemical termed DMBA diluted in olive oil was administered at a single dose of 80 mg/kg body weight (BW) through oral administration. A total of 30 rats were randomly assigned into five groups (n = 6 per group). Group I received physiological saline (control); Group II received DMBA; and Groups III, IV, and V received, in addition to DMBA, SrR on a daily basis at doses of 10, 100, and 200 mg/kg BW, respectively. The treatment lasted 14 weeks. During the evaluation, the time to the appearance of the mammary tumors (latency) was controlled by palpations in the mammary zone, and the BW of the rats was recorded weekly. At the end of the experiment, the animals were sacrificed by pentobarbital overdose.

All tumors were counted in each rat, and then removed to determine their volume and histopathological analysis. The cumulative tumor volume was calculated by the formula:


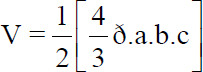


Where: V= volume; a = width; b = length; c = height.

### Histological analysis

After the sacrifice of the rats, the mammary tumors were removed and washed with physiological serum. The total number of tumors and the cumulative tumor volume (cm^3^) was determined [19]. For the histopathological study, 0.5×1.0 cm-thick sections were prepared and fixed in 10% neutral formalin; moreover, and microtome sections with a thickness of 3 mm were obtained. Finally, the sections were stained with hematoxylin and eosin (H&E) for evaluation of necrosis, mitosis (mild, moderate, and pronounced), and infiltration using an optical microscope (Olympus BX51, Tokyo, Japan).

### Acute toxicity in mice

The acute single-dose toxicity of SrR was assessed according to the 423 guidelines given by the OECD. Three BALB/c female mice were used in each step. The dose level that was used as the starting dose was 300 mg/kg BW. One group was used as the negative control, which was administered 2 mL/100 g BW of 3% polysorbate 80 orally, while the other treatment groups received SrR orally at different doses: 300 and 2000 mg/kg, respectively. At the beginning of the experiment, the weight of each animal was determined and mice were observed for 4 h (to check for toxicity signs after the treatment); subsequently, the mice were observed once a day for a period of 14 days, during which changes in behavior and other parameters, such as BW, food intake, motor activity, tremor, diarrhea, eye and skin colors, and death, were monitored [20].

### Absorption, distribution, metabolism, excretion (ADME) and molecular property prediction of the main volatile phytochemicals of SrR

A theoretical *in silico* ADME prediction study, under Lipinski’s “Rule of five” [21], was employed based on the main phytochemicals with high values (expressed as percentages), that is, Hop-22(29)-en-3b-ol, 1,4-benzenediol mono-tetradecyl ether, and cis-totarol, methyl ether. Lipinski’s parameters were calculated using a free web tool, Swiss ADME [22]. Other parameters, such as topological polar surface area, water solubility, CYP2D6, CYP2D9, P-glycoprotein inhibition, and phospholipidosis induction, were determined.

### Statistical analysis

The study parameters, such as total number of tumors and cumulative tumor volume (cm^3^), were expressed as absolute values, as well as averages and standard deviations. The incidence of tumors and histopathological findings, such as necrosis, mitosis, and inflammation, was expressed as percentages. The data were analyzed to determine the homogeneity of the variance using the Levene test and to assess normality using the Wilk−Shapiro statistics. A one-way analysis of variance was performed, followed by the Tukey multiple comparison method, to compare the average value of the different groups. Fisher’s exact test was used to assess differences in the incidence of tumors, necrosis, mitosis, and inflammation. Significance was set at p<0.05 in all cases. Data were analyzed using SPSS v. 21.

## Results

### Phytochemical analysis

**Figure-1:**
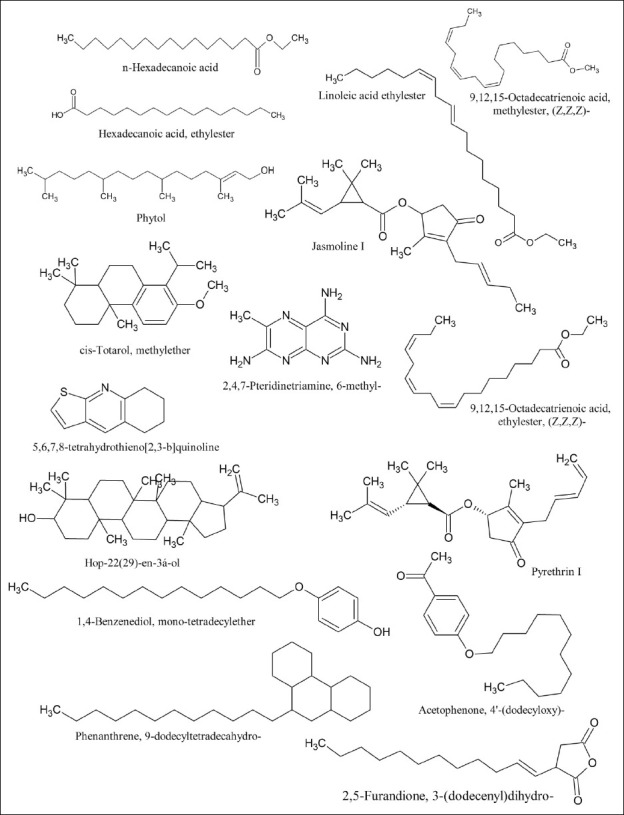
Main phytochemicals identified in the ethanol extract of *Senecio rhizomatus* Rusby by Gas Chromatography coupled to Mass Spectrometry.

The GC-MS analysis revealed the presence of cis-totarol methyl ether (3.93%), 1,4-benzenediol, mono-tetradecylether (44.18%), and Hop-22(29)-en-3β-ol (4.24%) as the major volatile components of the aerial parts of SrR (Figure-1). As shown in Table-1, several components were not identified that represented about 35% of the total components of the extract.

**Table-1:** Compounds identified in ethanol extract of *Senecio rhizomatus* Rusby aerial parts.

n	Compounds	IR^exp^	IR^ref^	Percentage
1	n-Hexadecanoic acid	1958	1957	0.68
2	Hexadecanoic acid, ethylester	1992	1991	0.47
3	Phytol	2109	2122	0.51
4	9,12,15-Octadecatrienoic acid, methyl ester, (Z, Z, Z)-	2135	2098	0.64
5	Unidentified	2148	-	0.34
6	Linoleic acid ethyl ester	2160	2155	0.57
7	9,12,15-Octadecatrienoic acid, ethyl ester, (Z, Z, Z)-	2166	2153	1.01
8	Unidentified	2193	-	1.69
9	Jasmoline I	2208	2345	0.26
10	cis-Totarol, methyl ether	2232	2223	3.93
11	5,6,7,8-Tetrahydrothieno[2,3-b] quinoline	2329	-	1.23
12	2,4,7-Pteridinetriamine, 6-methyl-	2395	2255	0.66
13	Pyrethrin I	2417	2335	1.34
14	1,4-Benzenediol, mono-tetradecyl ether	2439	2382	44.18
15	Unidentified	2467	-	0.98
16	Phenanthrene, 9-dodecyltetradecahydro-	2489	2638	0.42
17	Unidentified	2503	-	0.87
18	Acetophenone, 4’- (dodecyloxy)-	2512	2312	1.03
19	Unidentified	2524	-	19.43
20	Unidentified	2533	-	9.42
21	Unidentified	2614	-	0.96
22	2,5-Furandione, 3-(dodecenyl) dihydro-	2634	2159	0.64
23	Unidentified	2647	-	4.50
24	Hop-22 (29)-en-3β-ol	2668	2848	4.24
				100.00

LRIexp, Linear Retention Index calculated against n-alkanes C9-C24; LRIref, Linear Retention Index obtained from the literature NIST

### Antioxidant activity of ethanol extract of SrR, as assessed using the DPPH method

Table-2 shows the antioxidant activity of the ethanol extract of SrR, as assessed using the DPPH method, with a value (in percentage) that was near that of the control substance, that is, Vitamin C. A value of 100% of free radicals was taken as 0.8 at 517 nm using an ultraviolet−visible spectrophotometer.

**Table-2:** Antioxidant activity of the ethanolic extract of *Senecio rhizomatus* Rusby against DPPH radical.

Samples	Median value (n=3)	Standard error	Confidence interval 95%		Percentage

			Lower limit	Upper limit
DPPH	0.80	0.03	0.73	0.86	0.00
SrR 200	0.12	0.01	0.08	0.16	92.50
VitC 200	0.13	0.02	0.05	0.22	99.75

SrR 200=*Senecio rhizomatus* at 200 ug/mL dissolved in DMSO; VitC 200 = A solution of vitamin C at 200 ug/mL dissolved in distilled water, DPPH=1,1-Diphenyl-2-picril-hidrazil

### Evaluation of the protective effect of SrR on DMBA-induced BC in female rats

#### Histopathological evaluation

As shown in Table-3, the groups that received SrR exhibited a lower number of tumors on average, as well as a lower cumulative tumor volume compared with the DMBA group (p*<*0.05). The incidence of tumors, which was expressed as the ratio of affected rats to the total number of animals, was 100% in the DMBA compared with the DMBA + SrR 100 and DMBA + SrR 200 groups 50% each (p*<*0.05). The tumor latency, which was interpreted as the number of days until the appearance of the tumors, was, on average 47.3±1.96 days in the DMBA group, and was higher in the groups that received SrR 10, 100, and 200 mg/kg (56.3±1.50, 53.6±1.50, and 50.6±1.51 days, respectively) (p*<*0.05). At the histopathological level, the groups treated with SrR showed 0%–16.67% of necrosis, as well as light mitosis (100.00%), unlike the DMBA group, which exhibited necrosis in 66.67% and moderate mitosis in 100.00% of the animals.

**Table-3:** Effect of *Senecio rhizomatus* Rusby (SrR) on histopathological parameters.

Experimental group	Negative control	DMBA	DMBA + SrR10	DMBA + SrR100	DMBA + SrR200
Total tumors (N)	0	16	9	8	9
Tumors (mean ± SD)	0	2.67 (0.51)	1.5 (0.54)^α^	1.33 (0.51)^α^	1.50 (0.54)^α^
Volume of the tumor (mean ± SD)	0	0.59 (0.02)	0.73 (0.03)	0.34 (0.02)^α^	0.34 (0.02)^α^
Cumulative tumor volume (cm^3^)	0	9.45	6,65	2,72	3.08
Rats with tumors/Total	0/6	6/6	4/6	3/6	3/6
Tumor incidence (%)	0	100.00	66.67	50.00^β^	50.00^β^
Latency tumors (mean ± SD)	0	47.3 (1.96)	56.3 (1.50)^α^	53.6 (1.50)^α^	50.6 (1.51)^α^
Histopathology					
Necrosis (%)	0/6 (0)	4/6 (66.67)	1/6 (16.67)	0/6 (0)^β^	0/6 (0)^β^
Mitosis light (%)	0/6 (0)	0/6 (0)	6/6 (100.00)^β^	6/6 (100.00)^β^	6/6 (100.00)^β^
Moderate mitosis (%)	0/6 (0)	0/6 (100.00)	0/6 (0)^β^	0/6 (0)^β^	0/6 (0)^β^
Infiltration (%)	0/6 (0)	6/6 (100.00)	6/6 (100.00)	6/6 (100.00)	6/6 (100.00)

Negative control = Physiological saline, 2 mL/kg; DMBA = 7, 12-Dimethylbenz [a] anthracene 20 mg/kg; SrR 10, SrR 100 and SrR 200 = 10 mg/kg, 100 mg/kg, and 200 mg/kg of SrR, respectively. The presence of necrosis, mitoses, and infiltration was expressed in the number of rats on the total. α=Tukey test (p<0.01) versus DMBA. β=Fisher’s exact (p<0.01) versus DMBA

**Figure-2:**
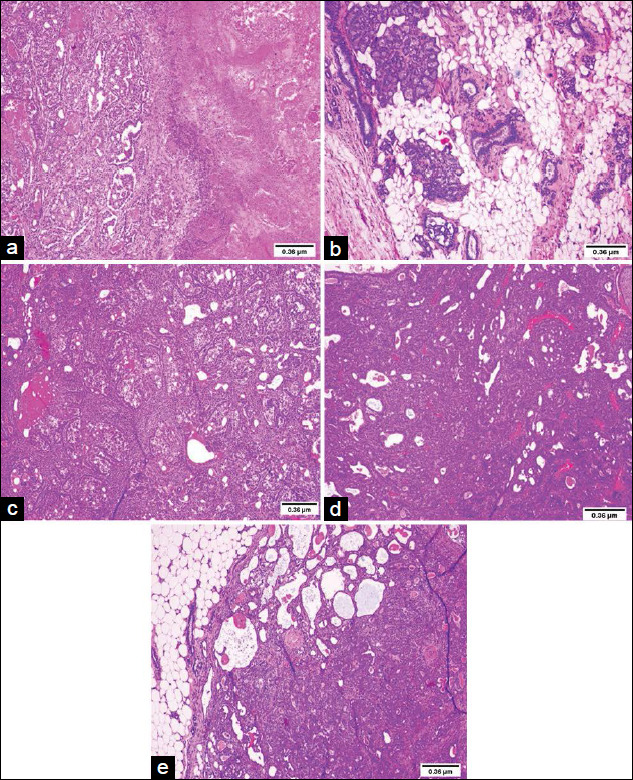
Staining of hematoxylin and eosin of the mammary glands of control and experimental animals (40×): (a) Negative control: Negative control group. Breast tissue within normal limits. (b) 7, 12-Dimethylbenz[α]anthracene (DMBA) Group: Infiltrating carcinoma with extensive tumor necrosis. (c) DMBA+SrR 10: Solid infiltrating carcinoma with areas of necrosis. (d) DMBA+SrR 100: Solid infiltrating carcinoma with cribriform areas. (e) DMBA+SrR 200: Solid carcinoma with cribriform areas, infiltrating adipose tissue.

The sections stained with H&E showed a normal structure in the negative control group. The DMBA group presented infiltrating carcinoma with extensive tumor necrosis. In contrast, solid infiltrating carcinoma with areas of necrosis was observed in the DMBA + SrR 10 group and solid infiltrating carcinoma with cribriform areas was detected in the DMBA + SrR 100 and DMBA + SrR 200 groups (Figure-2).

### BW variations in animals treated with SrR

**Figure-3:**
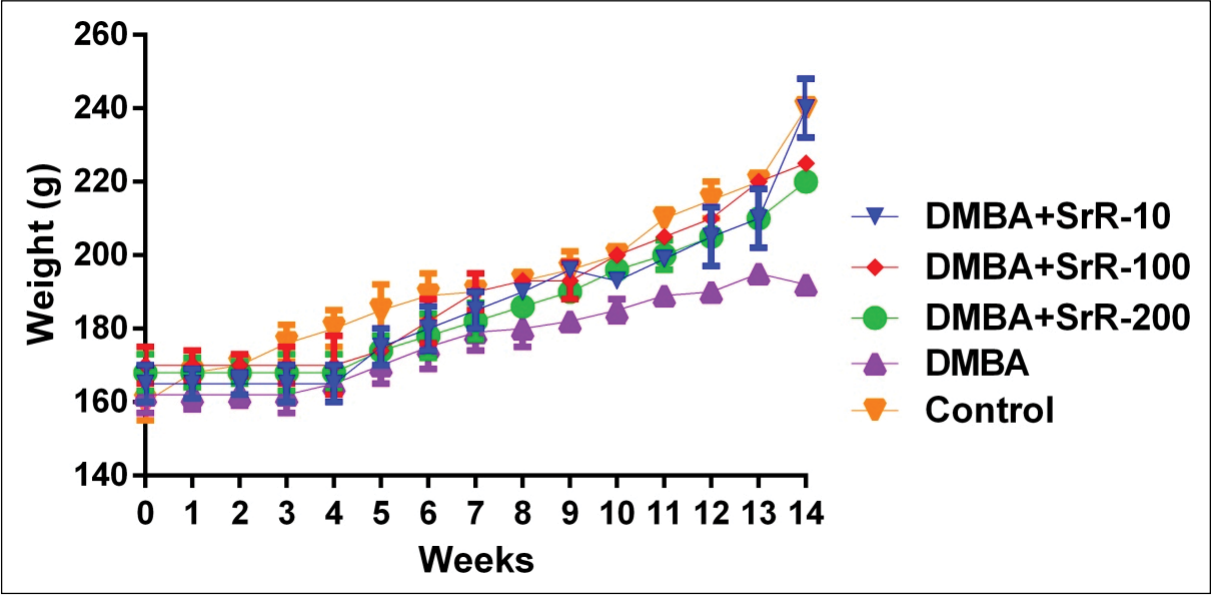
Weight variations of female rats during the evaluation of experimental breast cancer.

The BW of the female rats was controlled every week. A significant decrease in BW gain was observed in the DMBA group (p*<*0.05), starting at the 8^th^ week of treatment; the DMBA + SrR 100 mg/kg/day; and DMBA + SrR 200 mg/kg/day groups exhibited weight gain until the end of the evaluation (Figure-3).

### Evaluation of the acute toxicity of SrR in mice according to the OECD 423 protocol

The oral administration of SrR at doses of 300 and 2000 mg/kg did not induce death, and no signs of toxicity were observed after treatment up to 4 h of observation. Moreover, up to day 14, there were no changes in behavior and other parameters, such as BW, food intake, motor activity, tremor, diarrhea, and eye and skin colors.

### ADME and molecular property prediction

Bioavailability is an important factor in the therapeutic effect of orally administered drugs. The theorical predictors for the three compounds (­Hop-22(29)-en-3β-ol; 1,4-benzenediol-mono-tetradecylether, and cis-totarol, methyl ether) included by molecular flexibility, a poor intestinal absorption, a low polar surface area, poor solubility, and hydrogen-binding capacity. None of the phytochemicals passed Lipinski’s “Rule of 5” (Table-4). The Lipinski rule was violated regarding the MLogP values for the three compounds, as the values were >4.15. Moreover, 1,4-benzenediol-mono-tetradecylether showed inhibition of CYP1A2 and CYP2C19.

**Table-4:** ADME prediction of the volatile phytochemicals found in *Senecio rhizomatus* Rusby aerial parts.

Parameters	Hop-22 (29)-en-3β-ol	1,4-Benzenediol, mono-tetradecyl ether	cis-Totarol, methyl ether
Physicochemical properties			
Formula	C_30_H_50_O	C_20_H_34_O_2_	C_21_H_32_O
Molecular weight (≤500)	426.72 g/mol	306.48 g/mol	300.48 g/mol
Num. heavy atoms	31	22	22
Num. arom. heavy atoms	0	6	6
Num. rotatable bonds (≤10)	1	14	2
Num. H-bond acceptors (≤10)	1	2	1
Num. H-bond donors (≤5)	1	1	0
Molar refractivity	135.14	97.45	96.10
TPSA (≤140 Ǻ^2^)	20.23 Å²	29.46 Å²	9.23 Å²
Lipophilicity			
Log *P*_o/w_ (MLOGP) ≤4.15	6.92	4.65	5.14
Water solubility			
Log *S* (Ali)	−10.22	−8.82	−7.05
Class	Insoluble	Poorly soluble	Poorly soluble
Pharmacokinetics			
GI absorption	Low	High	Low
BBB permeant	No	No	No
P-gp substrate	No	No	Yes
CYP1A2 inhibitor	No	Yes	No
CYP2C19 inhibitor	No	Yes	Yes
CYP2C9 inhibitor	No	No	Yes
CYP2D6 inhibitor	No	Yes	No
CYP3A4 inhibitor	No	No	No
Druglikeness			
Lipinski rule [21]	Yes; 1 violation: MLOGP>4.15	Yes; 1 violation: MLOGP>4.15	Yes; 1 violation: MLOGP>4.15

BBB=Blood–brain barrier, GI=Gastrointestinal, P-gp=Glycoprotein P, TPSA=Topological polar surface area, ADME=Absorption, distribution, metabolism, excretion

## Discussion

The ethanolic extract of *Sr*R contained volatile components, as assessed by GC-MS, being the first SrR phytochemicals identified to date. Other reports of *Senecio* genera, such as *S. scandens*, revealed the presence of phenolic acids, flavonoids, saponins, lactones, terpenes, carotenoids, and volatile chemicals [23]. Phenolic compounds, specifically flavonoids, have been described as chemopreventive agents in cancer therapy; quercetin, a type of flavonol, has been reported as an anticancer substance against prostate and BCs [24]. Moreover, it exhibits antioxidant, anti-inflammatory, and antitumor properties. Quercetin induces apoptosis in BC cells by suppressing the p38MAPK pathway [25]. The biochemical mechanisms and genotoxicity of this compound should be studied further. The cytotoxic effects of *S. graveolens* and 4-hydroxy-3-(3-methyl-2-butenyl) acetophenone isolated from this plant were tested in the BC cell lines ZR-75-1, MCF-7, and MDA-MB-231. The anticarcinogenic activity of the whole-plant extract was stronger than that of its major component [26].

Table-1 shows that the use of ethanol as a solvent yielded a greater amount of the 1,4-benzenediol mono-tetradecyl ether chemical component isolated from SrR, which may have contributed to the protective effect [27,28] because of its hydroquinone-related molecule (HQ; 1,4-benzenediol), a hydroxylated benzene metabolite with several biological activities, including antioxidant, neuroprotective, immunomodulatory, and anti-inflammatory functions. However, the anticancer activity of HQ is not well known, but has been investigated *in vitro* and *in vivo* in several cancer cells and models. HQ significantly induced the death of A431 (human squamous carcinoma), SYF (mouse embryonic fibroblasts), B16F10 (mouse melanoma), and MDA-MB-231 (human cancer) cells; showed a synergistic effect with other antitumor agents and suppressive effect on angiogenesis in fertilized chicken embryos; and prevented lung metastases from melanoma cells in mice in a dose-dependent manner without toxicity and adverse effects. HQ (10 mg/kg) prevented colon cancer induced by sodium azoxymethane/dextran sulfate administration in mice [29].

As indicated in Table-1, the ethanolic extract of SrR contains a chemical agent called totarol, which has antimicrobial, antioxidant, and antitumor effects. Studies showed that totarol is the main component of the *Kaempferia parishii* rhizome extract and possibly responsible for the antitumor effect of the plant. Conversely, a phytochemical termed Hop-22(29)-en-3β-ol was found at a lower amount in SrR. A study of *Cnidoscolus chayamansa* isolated a chemical compound called moretenol (Hop-22(29)-en-3β-ol) that exhibited a high percentage of anti-inflammatory activity compared with the total extract [30]. An investigation of *Ficus deltoidea* extracts showed its pro-apoptotic and anti-migratory effects on the human prostate cancer cell line PC3, with the hexane fraction having the major effect; oleanolic acid, moretenol, betulin, lupenone, and lupeol were isolated from this fraction and were responsible for apoptosis by activating its intrinsic pathway [31].

The protective effect of SrR against BC in rats was evidenced by the lower number of tumors, tumor volume, and tumor incidence compared with the DMBA group; moreover, the time to tumor appearance was longer. The DMBA group presented infiltrating carcinoma with a higher incidence of necrosis and moderate mitosis, in contrast with the infiltrating carcinoma with cribriform areas observed in the DMBA + SrR 100 and DMBA + SrR 200 groups. Carcinomas are the most common among the breast tumors induced experimentally by DMBA [32], reaching 100%, of which approximately 50-90% are histologically malignant tumors. Tumor necrosis is rarely observed in Grade I carcinomas, in contrast to Grade III tumors, in which the disease is easily associated with some degree of infiltration; these characteristics are similar to those described for human ductal carcinoma. The micro cribriform pattern is mainly observed in Grade I carcinomas [33].

In the test of antioxidant capacity *in vitro*, the extract inhibited the DPPH radical at 92.5%, which may be because it contains phenolic compounds with a structure resembling that of a flavonoid. Flavonoids have been shown to have free-radical-scavenging properties through electron donation and to reduce the formation of lipid peroxidation by neutralizing the chain reaction in the formation of reactive oxygen species. Our data indicate the chemopreventive potential of the SrR extract in BC, with a high therapeutic index.

Based on ADME properties predicted *in silico*, the three main volatile components identified by GC-MS might not be responsible for the protective effect against BC progression; other components, such as alkaloids or flavonoids [34], could be involved, as reported in other *Senecio* species [34,35]. Moreover, in medicinal plants, it is known that a set of molecules are needed to generate a pharmacological effectand not necessarily the most representative molecule. Conversely, HPLC-MS may be necessary to identify the non-volatile components of the SrR extract, which was one of the limitations of this study, together with the lack of determination of the biochemical mechanisms involved in the effects of the extract.

## Conclusion

According to our results, the oral administration over 14 weeks of the ethanol extract of SrR had a protective effect on DMBA-induced BC in female rats, with changes observed in the histopathological study, as well as a reduction in the tumor cumulative volume and BW gain. The vitro antioxidant activity of the extract was not related with the protective effect because other biochemical parameters were not measured. Regarding the toxicity study, the extract was not toxic at the evaluated doses according to the OECD 423 guidelines, and three main phytochemicals were identified by GC/MS; however, we did not attribute the protective effects to their presence, and further studies are necessary to establish any biochemical mechanism and immunohistochemical analyses are necessary to assess BC progression.

## Authors’ Contributions

JLA and OH conceived the study designed. JLA and JPR performed the experiment. OH and RC analyzed the data. TB, JC, and OH drafted and revised the manuscript. All authors read and approved the final manuscript.
